# Endothelial Reprogramming in Atherosclerosis

**DOI:** 10.3390/bioengineering11040325

**Published:** 2024-03-27

**Authors:** Lu Zhang, Xin Wu, Liang Hong

**Affiliations:** 1 Department of Medicine, University of Illinois at Chicago, Chicago, IL 60612, USA; 2 Department of Biomedical Engineering, University of Illinois at Chicago, Chicago, IL 60612, USA; 3 Department of Physiology and Biophysics, University of Illinois at Chicago, Chicago, IL 60612, USA

**Keywords:** atherosclerosis, endothelial cell, reprogramming, EndMT, EndIT

## Abstract

Atherosclerosis (AS) is a severe vascular disease that results in millions of cases of mortality each year. The development of atherosclerosis is associated with vascular structural lesions, characterized by the accumulation of immune cells, mesenchymal cells, lipids, and an extracellular matrix at the intimal resulting in the formation of an atheromatous plaque. AS involves complex interactions among various cell types, including macrophages, endothelial cells (ECs), and smooth muscle cells (SMCs). Endothelial dysfunction plays an essential role in the initiation and progression of AS. Endothelial dysfunction can encompass a constellation of various non-adaptive dynamic alterations of biology and function, termed “endothelial reprogramming”. This phenomenon involves transitioning from a quiescent, anti-inflammatory state to a pro-inflammatory and proatherogenic state and alterations in endothelial cell identity, such as endothelial to mesenchymal transition (EndMT) and endothelial-to-immune cell-like transition (EndIT). Targeting these processes to restore endothelial balance and prevent cell identity shifts, alongside modulating epigenetic factors, can attenuate atherosclerosis progression. In the present review, we discuss the role of endothelial cells in AS and summarize studies in endothelial reprogramming associated with the pathogenesis of AS.

## 1. Introduction

Atherosclerosis (AS) is a chronic inflammatory disease of the large arteries and stands as a formidable challenge in the realm of cardiovascular diseases, and it accounts for approximately two-thirds of cardiovascular disease deaths globally, including coronary artery disease and ischemic atherosclerotic stroke. Its significant impact on global health is evidenced by its contribution to substantial morbidity and mortality [1]. In the United States, from 2015 to 2018, 38.1% of adults had total cholesterol levels ≥200 mg/dL, 27.8% had low-density lipoprotein cholesterol levels ≥130 mg/dL, 21.1% had triglyceride levels ≥150 mg/dL, and 17.2% had high-density lipoprotein cholesterol levels <40 mg/dL [2]. A study demonstrated that for every 10-point increase in average non-HDL-C levels above 125 mg/dL between the ages of 35 and 55 years, there was a corresponding 33% increase in the risk of future atherosclerotic cardiovascular disease [3]. AS places a tremendous burden on healthcare costs in society with frequent hospitalizations and associated morbidity and increased mortality, highlighting the effective strategies for the prevention and treatment of this disease.

AS is the most common form of cardiovascular disease characterized by lipid accumulation and inflammation of the large arteries. The wall of arteries consists of a monolayer of endothelial cells (ECs) that separate the blood from the vessel wall. Endothelial dysfunction is a hallmark and plays a crucial role in AS, which can be defined as a shift from a “calm” or non-activated state, characterized by low permeability and anti-thrombotic and anti-inflammatory properties, to an “activated” state, and by vasoconstriction and increased permeability and pro-thrombotic and pro-inflammatory properties [4]. Recent studies have identified that endothelial dysfunction is associated with transcriptional reprogramming in which endothelial cells shift toward mesenchymal cellular phenotypes and functional responses [5,6]. Endothelial-to-mesenchymal transition (EndMT) is a common and potentially pathophysiological process [7,8,9] and is widely involved in the occurrence and development of cardiovascular diseases [6,10,11]. During the process of EndMT, endothelial cells lose their cellular properties, such as the loss of tight junctions, and exhibit certain characteristics of mesenchymal cells, such as increased motility and increased secretion of extracellular matrix proteins. Recent studies in humans, porcine, and mice found that mesenchymal cells in atherosclerotic plaques derive from endothelial, indicating a link between EndMT and AS [12,13,14,15]. Additionally, EndMT facilitates leukocyte trafficking by reducing endothelial junctions [16]. Transforming growth factor-beta (TGF-β), a major inducible factor of EndMT, could increase the expression of ICAM-1 and damage vascular endothelial cell homeostasis [17]. In addition to immune response, single-cell analysis has confirmed that the increased overexpression of many pro-inflammatory genes and immune cell marker genes were from endothelial cells, indicating the potential acquisition of immune cell-like features (endothelial-to-immune cell transition; EndIT) [18]. The current review will discuss recent studies in endothelial reprogramming and atherosclerosis. The literature search was performed using OVID Medline, OVID EMBASE, and Pubmed, searching for “atherosclerosis”, “endothelial reprogramming”, “EndMT”, and “endothelial dysfunction”. Studies in the field will provide insights into novel therapeutic targets for the prevention and treatment of atherosclerosis.

## 2. Endothelial Activation

### 2.1. Physiological and Pathological Function of Endothelial Cells

**Figure 1:**
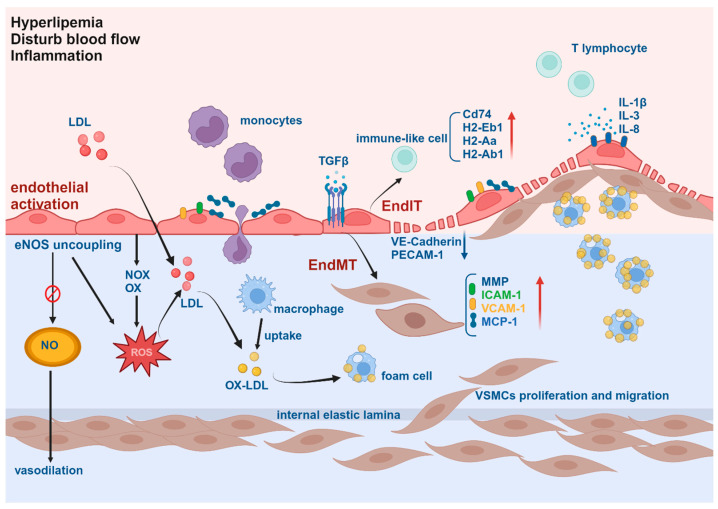
**Endothelial function in the development of atherosclerosis.** In the presence of hyperlipemia, abnormal blood flow, or inflammation, endothelial cells undergo a process known as “endothelial reprogramming”. This process involves endothelial transitioning from a quiescent, anti-inflammatory state to a pro-inflammatory and proatherogenic state. During this process, endothelial cells are activated, leading to eNOS uncoupling and increased ROS production associated with foam cell formation in atherosclerosis. Furthermore, this reprogramming is associated with phenotype alterations, including endothelial-to-mesenchymal transition (EndMT) and endothelial-to-immune cell-like transition (EndIT), both of which play a role in the progression of atherosclerosis. (Black arrow: activation; red arrow: upregulation; blue arrow: downregulation).

Endothelium, forming a semipermeable barrier along the vascular lumen, plays a crucial role in maintaining vascular homeostasis. In physiological conditions, it acts as a non-adhesive barrier to blood components while concurrently fulfilling various biological roles, including endocrine functions [4,19]. It can secrete many endothelium-derived bioactive substances, such as prostacyclin (PGI2) and nitric oxide (NO), as well as vasoconstricting substances, such as endothelin (ET-1), angiotensin II (Ang-II), and thromboxane A2 (TXA2). NO is known as endothelial relaxation factor (EDRF), a key vasodilator [20]. PGI2 and NO work synergistically to increase cAMP content in platelets, exhibiting robust vasodilatory, anti-leukocyte adhesion, and anti-platelet aggregation activities to maintain vascular homeostasis [21]. However, under the stimulation of pathological (e.g., disturb blood flow, high pressure, hypoxia) and chemical (e.g., oxidized low-density lipoprotein, free radicals, infection) factors, endothelial cells (ECs) are activated, and increase permeability, release cytokines to promote inflammation and thrombosis, and increase adhesion molecules to recruit and attach the inflammatory factor [22,23] (Figure 1). Endothelial activation has been regarded as a key role in both the initiation of the atherosclerotic process and the progression of advanced AS [23].

It is known that atherosclerotic plaques preferentially develop in a site-specific manner, especially at curved and branching regions of the arteries where there is disturbed blood flow (d-flow) [24,25,26]. Two animal models have been widely utilized in laboratories worldwide, serving as valuable tools for studying flow-dependent endothelial cell function and atherogenic mechanisms in vivo. One model involves partial carotid ligation (PCL) surgery, which induces d-flow in the left common carotid artery. In this procedure, three of the four caudal branches of the left common carotid artery are surgically ligated while leaving the artery itself intact. This results in characteristic low-magnitude oscillatory shear stress (OSS) patterns. Importantly, this model allows for an ideal comparison within the same animal, as the contralateral right carotid artery continues to be exposed to stable flow. Another model entails the implantation of a shear stress-modifying cast over a portion of a single carotid artery of hypercholesterolaemic Apoe^-/-^ mice with a Western diet. This cast exposes the portion of the artery proximal to it to low-magnitude stable flow, which induces the development of vulnerable plaques. Conversely, high-magnitude stable flow occurs within the casted region, resulting in less plaque development. Finally, disturbed flow is observed in the region distal to the cast, leading to the development of stable plaques. These models provide valuable insights into the effects of shear stress on plaque development and vulnerability [24]. The reason that d-flow induces AS may be because endothelial cells have the ability to sense and transduce the varied forces exerted by the pulsatile flow of blood into biological responses [24,27,28]. In brief, d-flow activates Nuclear Factor Kappa B (NFқB) and activator protein 1 (AP-1) in endothelia, leading to the upregulation of intercellular adhesion molecule-1 (ICAM-1) [29,30], interleukin (IL)-1a [31], bone morphogenic protein-4 (BMP-4), and monocyte chemotactic protein-1 (MCP-1) [32]. ICAM-1 not only increases the adhesion of T lymphocytes and ECs but also induces immune response by regulating the differentiation of B lymphocytes into plasma cells and producing antibodies [29,33]. MCP-1 can recruit circulating monocytes from the bloodstream into the intima, where they differentiate into macrophages [34], which together result in promoting inflammation infiltration. Consequently, endothelial activation promotes local inflammatory response, increases endothelial permeability, and decreases the ability to synthesize and secrete PGI2 and NO. In addition, endothelial activation switches NO signaling to reactive oxygen species (ROS) signaling, which could enhance inflammatory signals and oxidize the low-density lipoproteins (LDLs) to generate oxidized LDLs (oxLDL). Finally, macrophages phagocytose oxLDLs through scavenger receptors and transform into foam cells, which participate in the formation of atherosclerotic plaques [35]. The continuous accumulation of plaques and impaired homeostasis within the plaques can lead to lumen narrowing, plaque rupture, and blood overflow.

### 2.2. NO/ROS Redox Balance

#### 2.2.1. NO and NO Bioavailability

In addition to the function of substance exchange, transport, and innate immunity, ECs are the source of various cytokines, regulating vascular tone by balancing the production between vasodilators, including NO, PGI2, hydrogen sulfide (H2S), and vasoconstrictors, including ET-1 and Ang-II [36]. ECs regulate vascular hemostasis by secreting antiplatelet and anticoagulant molecules, such as NO, tissue plasminogen activator (t-PA), urokinase-type plasminogen activator (u-PA), tissue factor pathway inhibitor (TFPI), and thrombomodulin (Thbd) [27,37].

The loss of NO bioavailability in ECs is a typical alteration to response endothelial activation. NO is a potent endothelium-derived vasodilator and has multiple effects, such as vasodilation, antiplatelet, and antiproliferative. The ability of the endothelium to stimulate vasodilation was mediated by endothelium-derived relaxing factor (EDRF), known as NO [38,39,40]. Endogenous NO is produced through nitric oxide synthase (NOS) catalyzing the oxidation of L-arginine (L-arg) and oxygen. Acetylcholine, bradykinin, and calcium ionophores can stimulate NO gene and protein expression by regulating the transcription factors NF-κB and KLF [24,41]. There are three members in the NOS family, including neuronal NOS (nNOS) encoded by NOS1, inducible NOS (iNOS) encoded by NOS2, and endothelial NOS (eNOS) encoded by NOS3. eNOS is predominantly expressed in endothelial cells of blood vessels and endocardial cells, and it is also expressed in cardiomyocytes and platelets. A deficiency of eNOS was shown to reduce NO bioavailability. Transcription of the eNOS gene is regulated by fluid mechanical forces [42,43]. It has been reported that d-flow downregulated eNOS by activating the p90RSK-mediated SENP2-T368 phosphorylation pathway, leading to atherosclerosis (AS) [44]. A deficiency of eNOS exhibited heightened endothelial-leukocyte interactions, platelet aggregation, and thrombosis in mice [45]. The loss of eNOS can induce increased atherosclerotic plaques in the Apoe^-/-^ mice model of atherosclerosis [46]. It was reported that the eNOS function is crucial for the maintenance or the increase in NO bioactivity. The overexpression of eNOS reduced NO bioavailability in the endothelium, underlying the development of AS [47]. It was shown that eNOS produced superoxide rather than NO In the absence of its substrate L-arginine or tetrahydrobiopterin (BH4), and the inhibitory effects of BH4 on the development of AS were greater in the presence of eNOS overexpression [47]. BH4 is an essential cofactor for eNOS, and its oxidation straightly diminishes the availability, which subsequently leads to eNOS uncoupling [48]. The phenomenon of eNOS synthesizing superoxide instead of NO is referred to as eNOS uncoupling [49]. Superoxide removes NO, reduces its biology availability, and produces peroxynitrite, which can oxidize BH4 and the binding site of eNOS protein to the substrate L-arginine, resulting in further uncoupling of eNOS [50,51]. Additionally, eNOS uncoupling promotes the production and release of ROS, while ROS could oxidate BH4 and increase uncoupling, resulting in a vicious cycle.

#### 2.2.2. ROS

Reactive oxygen species (ROS) are a group of small reactive molecules, including superoxide, hydrogen peroxide, hydroxyl radical, and peroxynitrite, which serve as a signaling function to mediate biological processes. ROS signaling participates in normal physiological processes to maintain vascular homeostasis but also contributes to a maladaptive response that leads to endothelial dysfunction and inflammatory signaling. It plays a major role in the genesis of endothelial activation and barrier function [52] and oxidizes the LDL to generate oxLDL, initiating the foam cell formation and promoting AS progression [53,54,55]. ROS stimulates endothelial cells and macrophages to release adhesion factors and promote the production of MCP-1, thereby regulating monocyte invasion and inflammatory response in the lesion area. ROS in the large and middle arterial blood walls are mainly produced from the mitochondrial system through the electron transport chain (complex I to complex V) in the mitochondrial inner membrane during oxidative phosphorylation, nicotinamide adenine dinucleotide phosphate (NADPH) oxidase (NOX), xanthine oxidase (XO), and uncoupled eNOS [53].

##### NOX

NOX catalyzes the reduction in molecular oxygen (O_2_) to produce the reactive oxygen species superoxide. NOX expression has been detected in macrophages, vascular smooth muscle cells, endothelial cells, and fibroblasts, and the expression of NOX is related to the severity of AS. In mammalian cells, NOX has seven isoforms, of which NOX1, NOX2, and NOX4 are mainly expressed in the vasculature; the superoxide-generating enzymes are NOX1 and NOX2, and the hydrogen peroxide-generating enzyme is NOX4. NOX1 is involved in ROS generation, leukocyte adhesion, macrophage infiltration, vascular smooth muscle cell migration and proliferation, and extracellular matrix formation in AS. Increased NOX1 expression in the plaques of patients with cardiovascular disorders has been found, while the deletion of NOX1 reduces lesion area in the Apoe^-/-^ mouse model of AS [56]. Globe NOX2 deletion appeared to be protected from the production of ROS and atherosclerotic lesions in mice [57]. However, the role of endothelial NOX2 in the development of cardiovascular disease is controversial. Some studies found no link between pathological conditions and increased expression of NOX2, even though an increased production of superoxide and macrophage recruitment was observed. It seems like endothelial NOX2 is only involved in the initiation of AS, but the subsequent development of the atherosclerotic plaques is more relevant with immune cells [58,59], and macrophage NOX2 has been confirmed to be more detrimental in the development of cardiovascular disease [60]. The role of NOX4 in vascular biology appears to be generally protective [61,62]. This may be because NOX4 only generates hydrogen peroxide, but superoxide and hydrogen peroxide do not scavenge NO. The genetic deletion of NOX4 increases atherosclerotic plaque burden in the LDL^-/-^ mouse model of AS [62]. The endothelial-specific overexpression of NOX4 in mice promotes angiogenesis and eNOS function [63]. However, some studies found that NOX4 could promote fibrosis [64,65], which may be due to the increased activity of eNOS by hydrogen peroxide, which may induce eNOS uncoupling [66].

##### XO

XO is another resource to produce superoxide and hydrogen peroxide, and its activity increased with the increase in endothelial NOX activity. Studies have demonstrated that NOX could trigger eNOS uncoupling and activate xanthine under d-flow or hyperglycemia. Compared to the patients who have asymptomatic plaque, XO expression is significantly higher in symptomatic atherosclerosis patients [67]. XO could stimulate the expression of lectin-like oxidized low-density lipoprotein receptor-1 (LOX-1) and CD36 on macrophages and vascular smooth muscle cells, increase ROS production, and induce foam cell formation via the LOX-1/NLRP3 pathway [68]. Additionally, serum uric acid is also associated with atherosclerosis, which is the production of XO reactions. Serum uric acid may promote carotid plaque vulnerability by inducing mitochondrial dysfunction, ROS production, and inflammasome activation [69].

##### eNOS Uncoupling

eNOS uncoupling contributes to endothelial dysfunction, which consumes the equivalents to produce superoxide to aggravate oxidative stress, but no longer generates NO, thus inducing the development of AS. BH4 is the essential cofactor for the NO synthesis by eNOS and acts as the “NO/ROS redox switch” in eNOS uncoupling. ROS could oxide BH4 to generate dihydrobiopterin (BH2), which triggers eNOS uncoupling. eNOS is sensitive to imbalances in the concentrations of BH4 and BH2 because it has almost identical binding affinities for BH4 and BH2 [70,71]. Thus, rather than the relative amount of BH2 or BH4, the BH4/BH2 ratio gives a more precise estimation of NO bioavailability. BH4 is mainly synthesized by GTP-cyclohydrolase-1 (GTPCH), which is degraded upon oxidative activation of the 26S proteasome, and hyperglycemia-induced ROS production activates the 26S proteasome, leading to the ubiquitination and degradation of GTPCH [72]. Moreover, superoxide can form peroxynitrite and oxidize eNOS cofactor BH4 to cause BH4 deficiency, and then eNOS is uncoupled, increases superoxide production, and decreases NO production [73]. All these observations suggested that a deficiency in endothelial NO bioavailability will produce ROS, while ROS could further damage endothelial bioavailability [53,71,74,75], which leads to the occurrence and deterioration of AS in both humans and animals.

## 3. Alteration of Endothelial Cell Identity

### 3.1. EndMT

**Figure 2:**
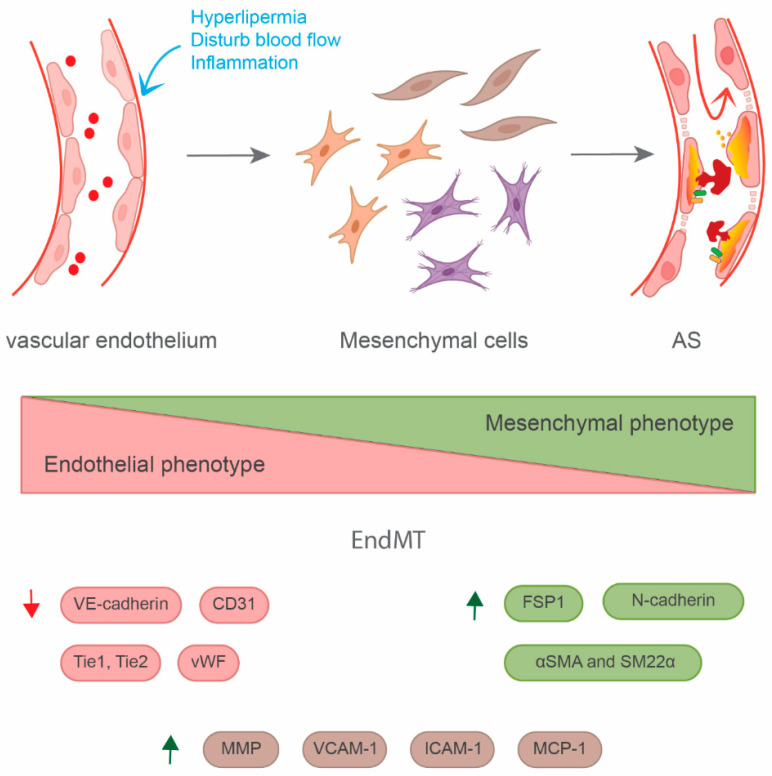
**EndMT in atherosclerosis.** In the presence of risk factors (e.g., hyperlipemia, d-flow, or inflammation), endothelial cells have the potential to differentiate into mesenchymal cells (such as smooth muscle cells, fibroblasts, and myofibroblasts). During this process, endothelial markers (VE-cadherin, CD31, Tie1/2, vWF) are downregulated, while mesenchymal cell markers (FSP-1, N-cadherin, α-SMA, SM22α) are upregulated. Additionally, endothelial cells lose adherent junctions, increase vascular permeability, and promote ICAM-1, VCAM-1, and MCP-1 expression to increase infiltration of inflammatory cells in atherosclerosis, along with increased MMP expression promoting plaque instability and accelerate plaque rupture during atherosclerosis.

EndMT was initially described in the endocardial cushions of the embryonic heart, and then it was speculated that it may persist at a low level throughout life [76]. However, recent evidence suggests that EndMT is crucial for the initiation and progression of cardiovascular diseases, such as cardiac fibrosis [6,10] and AS [11,15,77]. EndMT is an intricate cellular differentiation process whereby endothelial cells loosen their cobblestone-like and well-structured cell–cell junctions, degrade the basement membrane, and migrate out into the perivascular surroundings, and at the same time, decrease their properties and acquire mesenchymal features, characterized as the expression of vascular endothelial (VE)-cadherin, platelet/EC adhesion molecule-1 (CD31/PECAM-1), tyrosine kinase with immunoglobulin-like and epidermal growth factor (EGF)-like domains 1 (TIE1), TIE2, and von Willebrand factor (VWF), which are are diminished, while mesenchyme-specific factors, including N-cadherin, α-smooth muscle actin (α-SMA), smooth muscle protein 22α (SM22α), vimentin, fibronectin, and fibroblast specific protein-1 (FSP-1) are upregulated (Figure 2). A single-cell RNA-seq analysis of carotid arteries exposed to d-flow revealed that the carotid arterial endothelial cells reprogrammed into mesenchymal cells [18].

#### 3.1.1. EndMT Contributes to Inflammatory Infiltration

EndMT is regulated by several signaling pathways, such as bFGF/FGFR1, TGFβ/RSMADs, WNT/β-Catenin, PDGFR, and the hypoxic HIF1α signaling pathway, among which the role of the TGFβ/RSMADs signaling pathway is the most important factor driving EndMT. Transforming growth factor-beta (TGF-β) is one of the cytokines involved in maintaining homeostasis among cells within a given environment [78] and it is also a major driver of EndMT under interleukin-1β stimulation and abnormal shear stress [79]. There are three TGF-β isoforms (TGF-β1, TGF-β2, and TGF-β3), and all could induce EndMT in cultured endothelial cells [80,81,82], of which TGF-β2 plays a prominent role. The activin receptor-like kinases 1 and 5 (ALK1 and ALK5) are two prominent type I receptors of TGF-β in endothelial cells. The signaling of TGF-β through ALK5 subsequently induces nuclear translocation and binding of activated SMAD2/3 to SMAD-binding elements (SBEs) in the proximal promotors of mesenchymal genes, which induces their expression and inhibits endothelial cell proliferation and facilitates EndMT [77,83]. A study has confirmed that inhibiting TGF-β-mediated EndMT is an effective therapeutic strategy in atherosclerotic mice. The study found that the loss of endothelial cell epsins results in increased fibroblast growth factor receptor-1 (FGFR1) expression, which inhibits TGF-β signaling and EndMT. Epsins directly bind ubiquitinated FGFR1 through their ubiquitin-interacting motif (UIM), leading to endocytosis and degradation of this receptor complex [84]. Inflammatory cytokines and d-flow both led to a reduction in endothelial FGFR1 expression and activated TGF-β signaling. Additionally, there was a strong correlation observed between the loss of endothelial FGFR1 expression, the activation of endothelial TGF-β signaling and EndMT, and the severity of coronary atherosclerosis in patients [15]. Fibroblast growth factor (FGF) is a growth factor known to play important roles in vascular integrity and endothelial proliferation [85,86], and it might be the best endogenous inhibitor of EndMT. Furthermore, endothelial TGF-β signaling can increase cell adhesion [17] and permeability [87], which could promote ICAM-1, VCAM-1, monocyte chemotactic protein 1, and pro-inflammatory protein plasminogen activator inhibitor-1 protein expression to increase migration of inflammatory cells in AS. Also, d-flow could induce EndMT, which inhibits the expression of VE-cadherin and results in loosened adherent junctions and increased vascular permeability [12].

#### 3.1.2. EndMT Increases Atherosclerotic Plaque Progression

Notably, endothelial lineage tracing highlights the significance of EndMT as a crucial mechanism contributing to the accrual of activated fibroblasts and cells resembling smooth muscle cells within atherosclerotic plaques. Three to nine percent of the intimal plaque fibroblast-like cells were endothelial derived and thus had undergone complete EndMT [14]. Fibroblasts and vascular smooth muscle cells play crucial roles in atherosclerosis by regulating inflammation, the extracellular matrix, collagen production, and atherosclerotic plaque structural integrity [88,89]. Studies have shown that vascular smooth muscle cells can undergo a phenotypic transformation into macrophage-like cells, foam cells, mesenchymal stem cells, osteoblast-like cells, and other types, thereby affecting plaque stability. A further study found that the thickness of the plaque fiber cap was negatively correlated with the number of “transformed” cells. The greater the number of “transformed” cells, the thinner the fiber cap [14]. Unstable plaques have the following characteristics: a large necrotic lipid core, a large amount of inflammatory cell infiltration, and thin fibrous caps. According to the pathological classification of AS plaques, there are six types, including I–VI. Among them, type V is a thick fiber cap based on a larger lipid core, which is still covered by normal endometrial tissue. When the lesions of type IV and type V appear as secondary pathological changes, such as rupture bleeding, hematoma, and thrombus, it is called type VI [90]. An autopsy showed that there were cells expressing both endothelial and mesenchymal markers in human aortic atherosclerotic plaques, and the number of EndMT “transformed” cells in type VI plaques was more than that in type V plaques [14]. These outcomes provide compelling evidence that EndMT contributes to the onset and progression of atherosclerotic lesions and is correlated with plaque instability. EndMT induces plaque instability by giving rise to cells with a gene expression profile intermediate between endothelial cells and conventional fibroblasts. These “transformed” cells exhibit distinct extracellular matrix composition, which displays increased matrix metalloproteinase levels and diminished collagen expression. Collagen content is one of the main markers of plaque stability [91], and matrix metalloproteinases are closely related to plaque instability, which could degrade collagen and accelerate plaque rupture [92,93]. EndMT increases plaque instability by disrupting the balance between collagen and matrix metalloproteinases. Additionally, the co-localization of endothelial and calcification-related proteins was found in atherosclerotic plaques of Apoe^-/-^ mice [94]. Vascular calcification is a common feature of AS and is associated with increased plaque burden and poor clinical prognosis. Thus, EndMT could accelerate atherosclerotic plaque progression, cause vascular sclerosis and plaque rupture, and lead to acute myocardial infarction, stroke, sudden cardiac death, and other unconscious cerebrovascular events.

### 3.2. EndIT

Endothelial cells represent one of the initial cell types to engage with microbial components circulating in the bloodstream. ECs modulate metabolic homeostasis, vascular hemodynamics, permeability, coagulation, and cell extravasation under physiological conditions. When endothelium is activated, its surface also quickly transforms to a pro-coagulant, pro-inflammatitory state, except is does not amplify the immune response by recruiting immune cells. Activated ECs can generate and secrete pro-inflammatory cytokines and chemokine, including IL-1β, IL-3, IL-5, IL-6, IL-8, IL-11, and IL-15 [95]. It has been reported that the expression of TLR2 and TLR4 was upregulated in human atherosclerotic lesions [96]. Toll-like receptors (TLRs) are a series of pathogen-associated molecular pattern receptors on dendritic cells. In chronic inflammation, endothelium plays a crucial role through interactions with specialized effector cells and functions as antigen-presenting cells (APCs) [97]. Upon exposure to stimuli such as hydrogen peroxide and interferon-gamma (IFN-γ), ECs can elevate the expression of major histocompatibility complex I (MHC I) and instigate the expression of major histocompatibility complex II (MHC II) [97,98]. More importantly, the reprogramming of blood endothelial cells to lymphatic endothelial cells, and vice versa, has been extensively documented through the action of the homeobox transcription factor Prox1T [99,100], which emphasizes endothelial cell plasticity. Additionally, recent studies comparing the translatomes and transcriptomes of distinct tissue-specific endothelial cells have highlighted their heterogeneity and plasticity as they adapt to the tissue-specific environment [101,102,103]. Single-cell analysis has confirmed that the increased overexpression of many pro-inflammatory genes and immune cell marker genes were indeed contributed by ECs and put forward the concept of EndIT. Under d-flow conditions, they observed an upregulation of genes in the E8 cluster of endothelial cells, coinciding with high expression levels in immune cells (Cd74, H2-Eb1, H2-Aa, and H2-Ab1). Pseudo-time trajectory analysis, along with additional assessments in differential gene expression and chromatin accessibility, affirmed that E8 cells exhibit a heightened expression of marker genes associated with EndIT (C1qa, C1qb, C5ar1, and Tnf) compared to E2 cells. This suggests a potential transition toward endothelial-to-immune cell-like transformation in response to disturbed flow [18]. Thus, in addition to recruiting inflammatory cells, endothelial cells may enhance the inflammatory response by straightly transforming into immune cells.

## 4. Therapeutic Strategies Based on Endothelial Reprogramming in AS

So far, various strategies have been developed to address atherosclerosis by targeting different aspects, including reducing lipid levels, administering antiplatelet therapy, and using anti-inflammatory drugs [1]. However, current treatments have limitations and are unable to fully address the complexities of atherosclerosis. These treatments fail to eliminate atherosclerotic lesions, stabilize plaques, or halt the progression of the disease. For instance, patients who achieve their low-density lipoprotein cholesterol (LDL-C) target through regular treatment may still experience life-threatening cardiovascular events associated with AS [104]. Additionally, antiplatelet therapy carries the risk of increased bleeding, raising a significant concern [105]. Given the critical role of endothelial cells in AS and their inherent plasticity, targeting the endothelial reprogramming process may represent a novel therapeutic strategy against AS.

### 4.1. Inhibition of the EndMT Pathway

Recent studies have demonstrated that reversing or inhibiting EndMT can ameliorate AS simultaneously. Epsins can bind ubiquitinated fibroblast growth factor receptor-1 (FGFR-1) through their ubiquitin-interacting motif (UIM), leading to the endocytosis and subsequent degradation of this receptor complex, which consequently significantly attenuates EndMT and the progression of AS [84]. GSK3β inhibition or the endothelial-specific deletion of GSK3β reduces atherosclerotic calcification [106], while phosphorylation of GSK3β could increase the stability of Snail and thus EndMT [77]. Tenascin-X inhibits EndMT by suppressing TGF-β signaling through its interaction with αvβ3 integrin, thereby preserving endothelial integrity and preventing the progression of atherosclerotic lesions [107]. Thus, reversible regulation of EndMT may regress the development of AS. It has been revealed that Endoglin, an accessory type III TGF-β receptor, plays a role in partially regulating the equilibrium between ALK1 and ALK5 activation. By promoting downstream Smad1/5/8 responses, it indirectly inhibits ALK5 signaling and, consequently, suppresses EndMT [108]. Additionally, vascular endothelial growth factor A (VEGF-A)-stimulated VEGF receptor (VEGFR)2 signaling could also inhibit EndMT; however, this process is counteracted by the sequestration of VEGF-A by VEGFR1, which prevents its interaction with VEGFR2 and, consequently, leads to EndMT [109], and these findings suggest plasticity of these cells. BMP7 is implicated as a negative regulator of EndMT, primarily through the activation of ALK2 alone, along with the associated Smad1/5/8 pathway [110].

miRNAs have been shown to inhibit EndMT in various tissues. For instance, miR-15a, miR-23b, and miR-199a were found to impair EndMT during heart development [111]. Additionally, miR-155 acts as a potent inhibitor of TGF-β-induced EndMT by suppressing RhoA expression [112], and miR-630 was shown to inhibit EndMT in heterotopic ossification by targeting Slug [113]. Due to their high diversity, miRNAs hold great potential for therapy; however, the development of miRNA treatment is still in the early stages of development [114]. Additionally, neutralizing antibodies or chemical inhibitors targeting molecules essential for EndMT represents a promising approach to impede this process. Numerous compounds have been evaluated for the modification of EndMT, most of which interfere with TGF-β signaling. For instance, the dipeptidyl peptidase-4 (DPP-4) inhibitor linagliptin blocks TGF-β2-induced EndMT by disrupting its interaction with integrin β1. Meanwhile, the RGD antagonist RGD-2 has been shown to reverse TGF-β1-induced EndMT, offering potential as an anti-fibrotic therapeutic agent [115]. Additionally, the ALK5 inhibitor SB-431542 inhibits EndMT in cultured endothelial cells [116], while dorsomorphin inhibits EndMT by targeting the kinase activity of a mutant ALK2 in fibrodysplasia ossificans progressiva (FOP) [117]. Moreover, kallistatin inhibits TGF-β-induced EndMT by upregulating eNOS and downregulating EndMT-promoting miR-21 [118].

### 4.2. Directional Regulation of Endothelial-to-Mesenchymal Stem Cell Transformation

Moreover, the evidence that mesenchymal stem cells (MSCs) could derive from EndMT, which can be programmed to differentiate into various cell types, supports that EndMT could be utilized in tissue engineering and holds promise for treating AS. One study observed that ectopic bone formation in FOP originates from vascular endothelium that gives rise to MSCs, which suggests a linage with endothelial [117]. Other data show that EndMT gives rise to MSCs associated with differentiation into various cell lineages [119]. In one in vitro study, it is observed that high glucose, a known risk factor for atherosclerosis, induces EndMT in human aortic endothelial cells, with MSCs appearing during this process [120]. MSCs, also referred to as multipotent stromal cells, are adult pluripotent cells with the characteristics of self-renewal ability, pluripotent differentiation potential, immunomodulation, tissue regeneration, anti-inflammation, and low immunogenicity. Thus, there are two possible approaches to alleviate atherosclerosis focused on EndMT-derived MSCs.

#### 4.2.1. Regulation of the Differentiation of MSCs

MSCs have emerged as promising therapeutic agents for a wide range of diseases [121,122,123]. Given the role of inflammation in both the initiation and progression of AS, the regulation of MSCs, which have the capability of low immunogenicity and inhibition of T cell proliferation and memory T cell responses, presents a new therapeutic approach to AS [124,125]. The protective effects of MSCs observed in animal AS models are due to their production of various anti-inflammatory factors. For instance, interleukin-10 (IL-10) secreted by MSCs can inhibit macrophage activation, MMP activity, and the production of pro-inflammatory cytokines [126]. Additionally, MSCs contribute to the mitigation of inflammation by reducing the production of pro-inflammatory cytokines, such as TNF-α, IL-1β, and IL-6 [127]. The reduced expression of NF-κB in atherosclerotic plaques following MSC transplantation [128] is in line with numerous other studies, demonstrating the ability of MSCs to inhibit the expression and activity of NF-κB [126,129]. Additionally, MSCs significantly reduce plasma cholesterol levels in treated mice, as evidenced by a decrease in VLDL levels after 5 weeks of treatment [130]. In MSC-treated mice, there is a notable decrease in lipoprotein lipase expression in the liver, resulting in reduced availability of free fatty acids for VLDL synthesis. The deficiency of lipoprotein lipase in macrophages reduces the uptake of VLDL or ox-LDL, thus attenuating AS [131]. More importantly, MSCs accelerate the endothelial repairing process, although ECs have the potential to self-repair in response to inflammatory stimuli. One study demonstrated that allogeneic bone marrow-derived mesenchymal stem cell (BM-MSC) transplantation attenuates AS through repairing the diseased endothelium and improving endothelial function [132]. BM-MSCs have been considered an effective cell-based therapy. However, the procedure for bone marrow aspirate is highly invasive for patients and is accompanied by a risk of infection. The identification of MSCs derived from EndMT suggests that MSCs could be present in atherosclerotic lesions, which unveiled a novel, more accessible source of MSCs. Preventing mesenchymal stem cell differentiation may become a prominent therapy in AS. Moreover, a recent study revealed the role of SOX10 in stem cell maintenance, indicating the possibility of inhibiting stem cell differentiation [133]. However, further studies are required to determine the mechanism of MSC differentiation in AS.

#### 4.2.2. Reprogramming EndMT-Derived MSCs for Directed Differentiation

Given the unique properties of MSCs, including their potential for multi-directional differentiation, it is possible to directly reprogram EndMT-derived MSCs to differentiate specific cell types, such as vascular endothelial cells, to potentially repair damaged endothelial cells and decelerate the progression of AS. Direct cell reprogramming involves the use of small molecules or lentivirally transduced transcription factors to alter cell morphology and function directly, bypassing the need for a pluripotent state. This process entails the upregulation of lineage-specific transcription factors, and recreating developmental conditions is essential for successfully converting cells from one lineage to another. One study discovered that human neonatal fibroblasts transduced with lentiviruses Oct4 and KLF4 can undergo transdifferentiating into functional endothelial cells capable of revascularization [134]. Interestingly, although these reprogrammed cells exhibited high expression levels of established endothelial cell markers, they notably lacked expression of NOS3, an enzyme responsible for NO production in mature human endothelial cells. Additionally, another study investigated factors contributing to decreased efficiency in the direct reprogramming of fibroblasts into cardiomyocytes via viral transduction. The study found that fibroblasts undergoing direct reprogramming split into two populations: a reprogrammed population and a refractory population. The refractory population reverted to the original fibroblast RNA expression profile, indicating a potential barrier to successful reprogramming [135]. However, EndMT-derived MSCs may provide a solution to this issue. Due to the capability of endothelial cells to transition into MSCs through EndMT, there are Phase I and Phase II clinical trials currently underway. These trials involve the use of genetically engineered human umbilical vascular endothelial cells (HUVECs) to enhance bone marrow transplant, improve tendon healing following surgery, and treat non-healing ulcers. Results from these trials are anticipated within the next few years (NCT04190862, NCT04057833) [136]. Some studies have demonstrated that MSCs can acquire the characteristics of cell ancestries both inside and outside limb–bud mesodermal tissues [137]. In the presence of the essential transcription factor ETV2, undifferentiated mesodermal cells can differentiate into endothelial cells [138,139]. However, in vitro experiments have largely failed to translate to successful outcomes in vivo. Before considering the use of viral vectors for therapy, researchers should address the risks associated with nonspecific integration, which can lead to deleterious mutations. Advancements in alternative technologies offer promising solutions for direct reprogramming without the drawbacks of viral vectors. One is involved in the use of small molecule compounds to induce transdifferentiating. Small molecules offer several advantages, including lower cost, better dose control, and avoidance of the immunogenicity associated with viral transduction. Using this approach, chemically induced functional cardiomyocytes have been generated from fibroblasts in both mice and humans [140,141]. One recent study revealed that human dermal fibroblasts were successfully transdifferentiated into endothelial cells. This was achieved by inducing innate immunity with a toll-like receptor 3 (TLR3) agonist. Subsequently, endothelial-specific growth factors, such as VEGF and BMP4, were administered, followed by 8-Br-cAMP to induce fibroblast-to-endothelial transition, all without the need for lentiviral transduction [142]. miRNAs delivered via lipid nanoparticles show promise for somatic reprogramming; however, their application toward endothelial reprogramming has not yet been explored [143]. Although fibroblasts are a common starting cell source for reprogramming, the success of this strategy may be attributed to the lower epigenetic barriers compared to other differentiated cells [141]. Directly reprogramming EndMT-derived MSCs holds greater promise for therapeutic applications. In conclusion, direct programming offers a versatile platform applicable to a wide array of diseases. Reprogramming EndMT-derived MSCs may address key barriers to the direct reprogramming of ECs and the efficient differentiation rate and provide a potential therapeutic strategy for AS.

## 5. Concluding Remarks and Future Directions

Endothelial plasticity suggests that the dynamic process of endothelial reprogramming represents a pivotal aspect in the context of atherosclerosis. The transition of endothelial cells from a quiescent, anti-inflammatory state to a pro-inflammatory and proatherogenic state, or the alterations of cell cellular identity and function (EndMT and EndIT) both underscore the intricate interplay between endothelial biology and atherosclerotic progression. The elucidation of this phenomenon has been significantly advanced through innovative techniques, like single-cell RNA sequencing and many analyses, revealing these underlying molecular mechanisms even though the validation and relevance, particularly the application of EndIT in atherosclerosis, remain to be determined. The exploration of endothelial reprogramming targeted EndMT-derived MSCs has provided novel insights into the therapeutic strategy of atherosclerosis. Further research is required to validate the proposed strategies and identify small molecules or pharmacological tools that can modulate EndMT and restore endothelial homeostasis. In conclusion, targeting endothelial reprogramming holds promise as a viable approach for the development of effective therapies aimed at combating atherosclerosis and its related complications.

## Abbreviations

SMC	smooth muscle cell
EndMT	endothelial-to-mesenchymal transition
EndIT	endothelial-to-immune cell-like transition
TGF-β	transforming growth factor-beta
TLRs	toll-like receptors
VCAM-1	vascular cell adhesion molecule-1
ICAM-1	intercellular adhesion molecule-1
FGF	fibroblast growth factor
IFN-γ	interferon-gamma
APCs	antigen-presenting cells
MHC I	major histocompatibility complex I
MHC II	major histocompatibility complex II
VE-Cadherin	vascular endothelial-cadherin
CD31/PECAM-1	platelet/EC adhesion molecule-1
vwf	von Willebrand factor
α-SMA	α-smooth muscle actin
SM22α	smooth muscle protein 22α
LDL	low-density lipoproteins
LOX-1	low-density lipoprotein receptor-1
ROS	reactive oxygen species
NO	nitric oxide
NOX	nicotinamide adenine dinucleotide phosphate (NADPH) oxidase
XO	xanthine oxidase
EDRF	endothelium-derived relaxing factor
IL-1a	interleukin-1a
BMP-4	bone morphogenic protein-4
MCP-1	monocyte chemotactic protein-1
ET-1	endothelin-1
Ang-II	angiotensin II
TXA2	thromboxane A2
PGI2	prostacyclin2
d-flow	disturbed blood flow
NFқB	Nuclear Factor Kappa B
AP-1	activator protein 1
